# Increased Antigen Specific T Cell Numbers in the Absence of Altered Migration or Division Rates as a Result of Mucosal Cholera Toxin Administration

**DOI:** 10.1371/journal.pone.0059934

**Published:** 2013-03-27

**Authors:** Maria Kaparakis-Liaskos, Michelle D. Tate, Jason D. Price, Martin Pearse, Odilia L. C. Wijburg

**Affiliations:** 1 Department of Microbiology and Immunology, The University of Melbourne, Parkville, Victoria, Australia; 2 Centre for Innate Immunity and Infectious Diseases, Monash Institute of Medical Research, Clayton, Victoria, Australia; 3 CSL Ltd, Parkville, Victoria, Australia; National Council of Sciences (CONICET), Argentina

## Abstract

Cholera toxin (CT) is a mucosal adjuvant capable of inducing strong immune responses to co-administered antigens following oral or intranasal immunization of mice. To date, the direct effect of CT on antigen-specific CD4^+^ T cell migration and proliferation profiles *in vivo* is not well characterized. In this study, the effect of CT on the migration pattern and proliferative responses of adoptively transferred, CD4^+^ TCR transgenic T cells in orally or intranasally vaccinated mice, was analyzed by flow cytometry. GFP-expressing or CFSE-labeled OT-II lymphocytes were adoptively transferred to naïve C57BL/6 mice, and mice were subsequently vaccinated with OVA with or without CT via the oral or intranasal route. CT did not alter the migration pattern of antigen-specific T cells, regardless of the route of immunization, but increased the number of transgenic CD4^+^ T cells in draining lymphoid tissue. This increase in the number of transgenic CD4^+^ T cells was not due to cells undergoing more rounds of cellular division *in vivo*, suggesting that CT may exert an indirect adjuvant effect on CD4^+^ T cells. The findings reported here suggest that CT functions as a mucosal adjuvant by increasing the number of antigen specific CD4^+^ T cells independent of their migration pattern or kinetics of cellular division.

## Introduction

Cholera toxin (CT) is a well characterized mucosal adjuvant capable of inducing strong immune responses to co-administered antigens [1] following intravenous, oral, intranasal or sublingual immunization [2]–[4]. CT is a multimeric protein produced by *Vibrio cholerae*, consisting of a pentameric ring of B subunits associated with a single A subunit. The CT-B subunit binds the GM1 mono-sialo ganglioside, which is found in high abundance on the luminal surface of intestinal epithelial cells [5], [6]. The binding of CT to the cell membrane mediates ADP-ribosylation of an adenylate cyclase regulatory protein [7], resulting in increased intracellular cAMP concentrations. The increase in cAMP levels leads to changes in ion channels, the accumulation of Na^+^ and Cl^−^ ions in the gastrointestinal lumen, and a massive flow of water and electrolytes from epithelial cells [8]. The toxicity of CT for humans is extreme, as 5 µg of orally ingested CT leads to the production of more than 1 liter of diarrhea [9].

CT is a strong mucosal adjuvant and is thought to facilitate luminal antigen transport across the epithelium by increasing gut permeability, allowing antigens to access the gut mucosal immune system and induce an immune response [10]. In this way, CT mediates increased antibody production [11] and induces cytotoxic T lymphocyte activity when co-administered with antigen via the oral [12], intranasal, and subcutaneous routes [13]. The oral co-administration of soluble antigen with CT promotes priming of CD4^+^ T cells [14], elicits systemic Th2 activity [15], [16] and enhances the proliferation of lymphocytes to antigen [14]. This results primarily in the development of a Th2-like immune response to co-administered antigens, with increased production of IL-4, IL-5 and IL-10, and provides help for B cells to produce IgG1, IgA and IgE antibodies [11], [17], [18]. Indeed, CD4^+^ T cells are essential for the development of antigen-specific antibody responses following mucosal administration of both antigen and CT [19].

More recently, the effect of CT on antigen presenting cells has been elucidated. Conventional DCs (cDCs) are essential for the development of antigen-specific antibody responses following oral vaccination with CT and antigen [20]. This is due to the direct effect of CT on cDCs, inducing their migration into the follicle-associated epithelium as early as one hour after encountering CT [20]. Furthermore, cDCs are required for priming of CD4^+^ T cells following nasal or oral administration of protein with CT [20]. This may be due to the ability of CT to induce the upregulation of costimulatory molecules such as B7.2 on B cells, macrophages and dendritic cells (DCs) [17], [21], [22] and B7.1, B7.2, OX-40 and HLA-DR molecules on human DCs that can prime naïve CD4^+^ T cells *in vitro,* driving their polarization towards a Th2 response [23]–[25]. The ability of CT to upregulate costimulatory molecules on DCs may function in unison with its ability to induce the expression of functional CCR7 and CXCR4 chemokine receptors on DCs, enabling them to migrate towards secondary lymphoid organs and present antigen to T cells [23].

Despite the plethora of studies describing the adjuvant effect of CT and its effect on DC migration, its effect on antigen-specific CD4^+^ T cell migration into non-lymphoid and secondary lymphoid tissue, and the kinetics of *in vivo* proliferation, have not been defined to date. In addition, the effect of CT administration via various mucosal routes on T cell migration and proliferation has not been examined. Studies examining the direct effect of CT on T cells may further explain its adjuvanticity and aid the development of an alterative CT-based adjuvant suitable for human use. This study utilized the adoptive transfer of transgenic T cells to study *in vivo* antigen-specific, CD4^+^ T cell responses in the presence of the mucosal adjuvant CT. The migration pattern, number of antigen-specific T cells and the kinetics of antigen-specific CD4^+^ T cell division in response to oral or intranasal administration of antigen and CT were examined. This study established that CT acts by increasing the number of antigen-specific T cells regardless of the route of vaccination. This antigen specific CD4^+^ T cell increase did not appear to be due to an alteration in the migration profile of antigen-specific T cells *in vivo,* nor to an alteration in the kinetics of cellular division. However, the overall increase in antigen-specific cell number may be the result of more efficient activation of DCs, leading to cell division by an increased number of the fixed pool of antigen-specific cells.

## Materials and Methods

### Ethics Statement

All animal experiments were approved by The University of Melbourne Animal Ethics committee and performed in accordance with the Prevention of Cruelty to Animals Act (1986) and the NHMRC Code of Practice for the Care and Use of Animals for Scientific Purposes.

### Mice

Six to nine week old C57BL/6, C57BL/6^gfp+/−^ (referred to as B6.GFP mice) [26], B6.OT-II (OVA-specific, MHC class II restricted TCR transgenic mice) [27] and B6.OTII^gfp+/−^ mice (this study) were bred and housed at the animal facility of the Department of Microbiology and Immunology, The University of Melbourne. B6.OTII^gfp+/−^ mice used in this study were generated by crossing transgenic homozygous B6.OT-II with C57BL/6^gfp+/−^. These mice were mated to obtain heterozygous F1 offspring with the genotype C57BL/6^gfp+/−^ OT-II^+/+^. All mice were housed under specific pathogen free conditions and fed sterile food and H_2_O *ad libitum*. Mice were euthanized by carbon dioxide asphyxiation. All mice were age and sex matched for each experiment.

### Preparation of Lymphocyte Suspensions from Spleen and Lymph Nodes

Spleens were gently passed through a 40 µm-gauge wire mesh, and erythrocytes were lysed by incubation in NH_4_Cl-Tris solution (pH 7.2) at 37°C for 5 minutes. The cells were collected by centrifugation and resuspended in Hanks Balanced Salt Solution (HBSS) supplemented with 2.5% (v/v) fetal calf serum (HBSS/2.5% FCS). Lymph node (e.g. brachial, mesenteric, cervical and paragastric lymph node) cell suspensions were prepared in HBSS/2.5% FCS by gently teasing whole tissues through a 40 µm-gauge wire mesh. Cells were collected by centrifugation and resuspended in HBSS/2.5% FCS.

### Peyer’s Patch (PP) Lymphocyte Preparations

PP lymphocytes were prepared as previously described [28]. In brief, PP were removed from the intestine and incubated with HBSS/0.25 M EDTA to remove epithelial cells. Treated PP were then minced with a scalpel blade and further incubated with 200 µg/ml collagenase D (Boehringer Mannheim, Mannheim, Germany) and 40 µg/ml DNase I (Boehringer Mannheim, Mannheim, Germany) for 45 minutes at 37°C with gentle agitation. Single cell suspensions of the digested PP were prepared by passing the tissues through a 40 µm-gauge wire mesh.

### Preparation of Intraepithelial (IEL) and Gut Lymphocytes

IELs were prepared using a modification of a method described previously [29]. In brief, the small intestine was dissected, the intestinal contents were flushed out, and PP and mesentery were removed. The intestine was cut longitudinally, then into 2 cm to 3 cm lengths and incubated with 5 mM EDTA (pH 7.2). After washing, remaining mesentery was removed with the aid of a dissecting microscope. The segments were incubated with 100 U/ml collagenase D, 1.5 µg/ml DNase-I, and 1.5 µg/ml soybean trypsin inhibitor (Worthington, NJ, USA) for 15 minutes at 37°C. This digested fraction contained the IEL. Undigested tissue was sedimented by centrifugation at low speed, and the IELs were recovered from the supernatant. The remaining intestinal segments were incubated for 1 to 2 hours with the collagenase mix until completely digested. Cells recovered from this fraction were termed the gut cellular preparation.

### Isolation of Gastric Mucosal Lymphocytes

Lymphocytes were isolated from the gastric mucosa of mice using the “ballooning” method as previously described [30]. PBS/5% (v/v) FCS (10 ml) was injected into the mucosa of the stomach, forcing the tissue to swell and rupture, consequently releasing lymphocytes. This “ballooning” was performed along the whole surface of the stomach. Lymphocyte suspensions obtained were passed through nylon mesh to remove tissue debris.

### Isolation of Nasal Associated Lymphoid Tissue (NALT)

Lymphocytes were isolated from the NALT of mice as previously described [31].

### Adoptive Transfer of CFSE-labeled Lymphocytes

Lymphocytes from donor B6.OTII^gfp+/−^ or B6.OT-II mice were prepared from renal, mesenteric, cervical, brachial, auxiliary and lumbar lymph nodes. Single cell suspensions of lymphocytes were prepared and OT-II lymphocytes were labeled with the fluorescent dye CFSE (Molecular probes, OR, USA) [32]. In brief, 10^7^ lymphocytes were resuspended in 1 ml of HBSS/0.1% (w/v) BSA with 1 µM/ml of CFSE. Cells were labeled at 37°C for 10 minutes, washed twice with HBSS/2.5% (v/v) FCS, and finally resuspended in HBSS. Mice were injected intravenously (i.v.) with 5×10^6^ B6.OTII^gfp+/−^ (referred to as GFP^+/−^ OT-II cells) or CFSE-labeled B6.OT-II cells (referred to as CFSE-labeled OT-II cells) in 200 µl PBS, unless otherwise stated.

### Intranasal Administration of Evans Blue

Evans blue dye (BDH, England) (0.3% w/v in PBS) was administered to Penthrane anaesthetised mice (Medical Developments Australia Pty Ltd, Australia) via a micropipettor fitted with a 200 µl pipette tip. Mice were held in an upright position, and either 12 µl or 20 µl of Evans blue dye was gradually applied to the nares, allowing mice to inhale the dye. Mice were euthanized either immediately, 1 hour, 4 hours or 24 hours after Evans Blue administration and the location of the dye was determined by examining the organs (oesophagus, trachea, lungs, stomach and intestine) *in situ*.

### Immunization of Mice

One day after intravenous (i.v.) adoptive transfer of CFSE-labeled OT-II cells or GFP^+/−^ OT-II cells into recipient C57BL/6 mice were anaesthetized by Penthrane inhalation (Medical Developments Australia Pty Ltd, Australia) and OVA and/or CT were administered orally by gavage. Mice were orally immunized with 10 µg of CT (Sigma, St Louis, MO, USA), 15 mg of OVA (Sigma, St Louis, MO, USA), or a mixture of both, made to 200 µl with PBS. For intranasal (i.n.) immunization, anaesthetized mice were immunized with a 12 µl volume of either PBS, 25 µg OVA, 5 µg CT or a mixture of 25 µg OVA and 5 µg CT, gradually placed onto their nares.

### Bone Marrow Derived Dendritic Cell (BMDC) Cultures

Bone marrow derived DC cultures were generated according to Lutz *et al*. [33]. In brief, single cell suspension of bone marrow obtained from the femurs of C57BL/6 mice were cultured in RPMI 1640 supplemented with 10% (v/v) FCS and 10% (v/v) GM-CSF supernatant (X63-GM-CSF, kindly provided by Professor F. Carbone, The University of Melbourne). After 7 days of culture, semi and non-adherent cells were collected and used for all DC experiments. Cells were labeled with anti-CD11c and anti-MHC II antibodies and analyzed using flow cytometry prior to use.

### In vitro Stimulation with Peptide Pulsed DCs

Antigen-loaded BMDCs were co-cultured with CFSE-labeled OT-II cells as previously described [34]. BDMCs (4×10^6^/ml) were incubated with CT (4.4 µg/ml), synthetic OVA_323–339_ peptide (1 µg/ml) or CT with OVA peptide (Chiron technologies, Clayton Vic, Australia) for 45 minutes at 37°C in RPMI (Gibco, USA) [35]. BMDC were washed three times and added to 96 well round-bottom plates at 5×10^3^ per well. Naïve, CFSE-labeled OT-II cells were cultured with BMDC at 5×10^4^ per well (NUNC, Denmark). At various time points, dilution of CFSE dye was analyzed using flow cytometry.

### Flow Cytometry

Cells were labeled with fluorescently conjugated antibodies (BD Biosciences, USA) to CD4 (Clone H129.19) and Va2 (Clone B20.1) to enable identification of OT-II cells or to CD11c (Clone HL3) and MHC II (I-A^b^ Clone 25-9-17) to label DC. When indication, cells were labeled with antibody specific for the adhesion molecule a4b7 (Clone DATK32). All samples were acquired using a FACS Calibur (BD Biosciences, USA). Non-viable cells were excluded from data acquisition on the basis of 0° and 90° scatter profiles, and by their ability to exclude propidium iodide. Data was analyzed using CellQuest™ software (BD Biosciences).

### Statistics

Data was analyzed using a two-tailed Student’s *t*-test or one-way ANOVA as indicated. Differences were considered significant when *P*<0.05, whereas *P*<0.01 represents greater statistical difference.

## Results

### CT does not Affect the in vivo Migration Pattern of CD4^+^ T Cells in the Absence of Antigen

**Figure 1:**
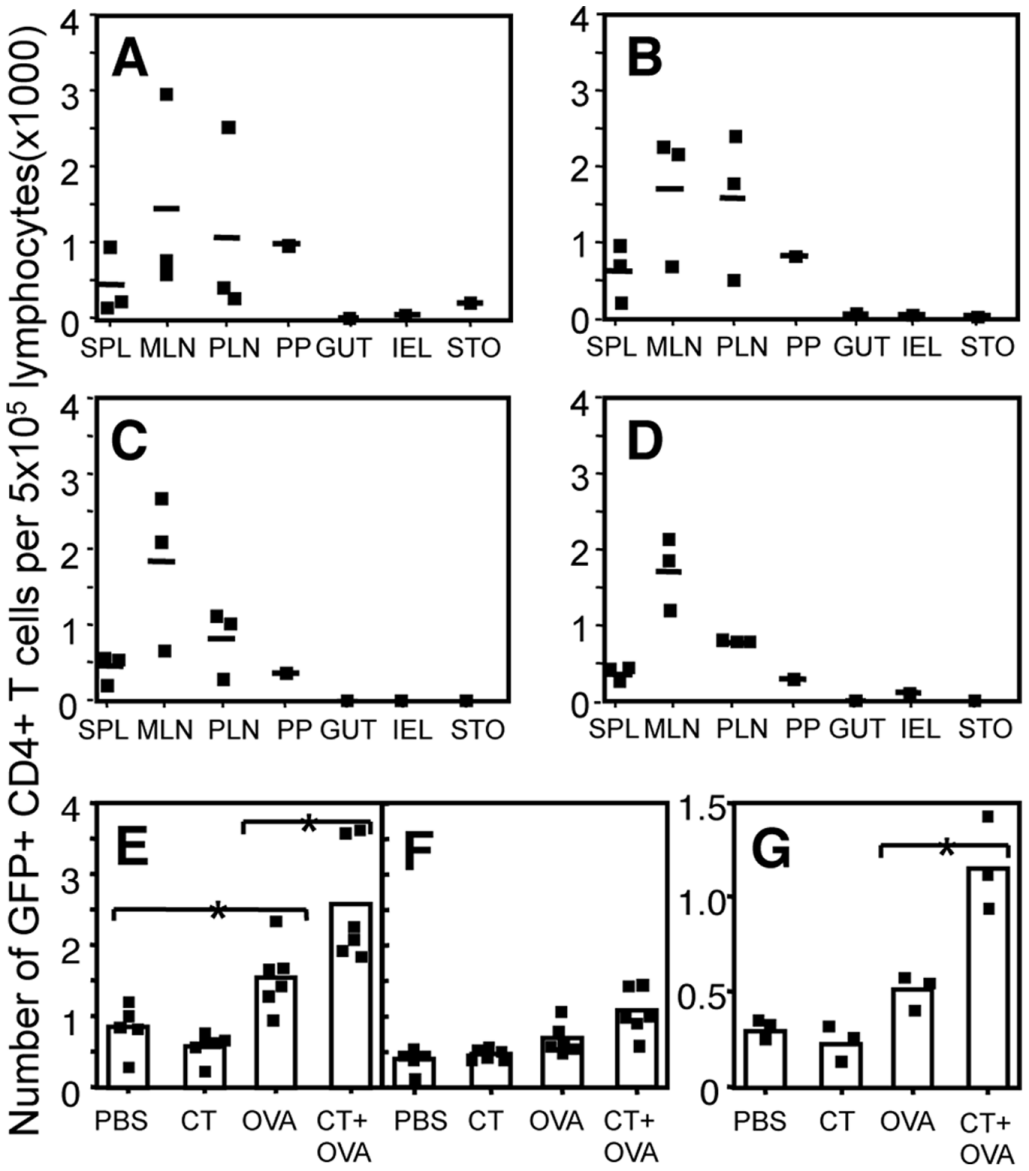
Oral administration of CT increases the number of antigen-specific CD4^+^ T cells without altering their migration pattern *in vivo*. GFP^+/−^ OT-II lymphocytes (5×10^6^) were adoptively transferred to C57BL/6 recipient mice and one day later the mice were orally immunized with PBS (A, C) or 10 µg CT (B, D). One (A–B) and five days (C–D) after oral immunization, the number of GFP^+/−^ OT-II cells (identified as GFP^+^CD4^+^) present in the spleen (SPL), mesenteric lymph nodes (MLN), peripheral lymph nodes (PLN), Peyers patches (PP), gut, intraepithelial lymphocyte compartment (IEL) and stomach (STO) was determined by flow cytometry. The lymphocyte preparations from the IEL, STO and PP of mice were pooled to obtain sufficient numbers of cells. Represented are the results from individual mice, as well as the average number of lymphocytes per group (dash, n = 3). Lymphocytes from the PP of mice were pooled (n = 3 each). Results are representative of one out of three independently performed experiments. E–G: GFP^+/−^ OT-II lymphocytes (5×10^6^) were adoptively transferred to C57BL/6 recipient mice. One day later, recipient mice were orally immunized with either PBS, 10 µg CT, 15 mg OVA or 15 mg OVA with 10 µg CT. Five days after oral immunization, the number of transgenic GFP^+/−^ OT-II (GFP, Vα2, CD4^+^) cells present in MLN (E), spleen (F) and PP (G) was determined by flow cytometry. E–F: Symbols represent results from individual mice obtained in 2 independently performed experiments, the columns represent the mean of each treatment group. G: symbols represent the results from three independent experiments each performed with PP cells pooled from 3 mice, the column represents the mean of the 3 experiments. *denotes *P*<0.05 (one way ANOVA).

To determine the effect of CT on the migration of antigen-specific T cells *in vivo*, GFP OT-II cells were adoptively transferred into recipient C57BL/6 mice which were orally immunized with either PBS (Figure 1A and C) or 10 µg CT (Figure 1B and D) the following day. At 1 and 5 days after oral immunization, the location of GFP^+/−^ OT-II cells in various organs was determined using flow cytometry (Figure 1). At both time points, GFP^+/−^ OT-II lymphocytes were detectable in all secondary lymphoid organs analyzed, and as expected, negligible numbers of GFP^+/−^ OT-II cells were detected in the (non-lymphoid) gut, stomach and in the lamina propria among intraepithelial lymphocytes (IEL) of PBS-control recipient mice. In comparison with PBS-fed mice, CT-immunized mice had similar numbers of GFP^+/−^ OT-II cells in lymphoid tissues and in non-lymphoid gastric tissue, suggesting that administration of CT did not alter the migration of antigen-specific T cells *in vivo* over time (Figure 1A–D).

### Oral Administration of CT with Antigen Increases the Total Number of Antigen Specific T Cells in vivo

We next examined if co-administration of CT with antigen affects the migration pattern or the number of antigen-specific T cells in the lymphoid tissues of mice. One day after adoptive transfer of GFP^+/−^ OT-II lymphocytes, recipient C57BL/6 mice were immunized orally with either 10 µg CT, 15 mg OVA, or 10 µg CT and 15 mg of OVA, or received PBS as a control. Mice were euthanized either on day 1, when no OT-II cell proliferation is evident, or day 5 after oral immunization, and the number of GFP^+/−^ OT-II T cells in secondary lymphoid organs was examined using flow cytometry (Figure 1E–G).

One day after feeding, there was no difference in the distribution or the number of transgenic GFP^+/−^ OT-II (Vα2 CD4^+^) T cells detected in any of the tissues of different groups of immunized mice (data not shown). However, 5 days after oral immunization, there was a significant increase in the number of GFP^+/−^ OT-II cells in the MLN of OVA immunized mice compared with PBS controls (*P*<0.05) (Figure 1E). Co-administration of CT with OVA further increased the numbers of GFP^+/−^ OT-II cells in the MLN compared with immunization with OVA alone (*P*<0.05). In the PP, the number of GFP^+/−^ OT-II cells significantly increased in the CT and OVA immunized mice compared with OVA alone (Figure 1G) (P<0.05, one way ANOVA), while no effect on GFP^+/−^ OT-II cell number was observed in the spleen of mice immunized with CT and OVA compared with OVA alone (Figure 1F). No GFP^+/−^ OT-II cells were detected in any non-lymphoid organs (stomach, IEL and gut) analyzed (data not shown). Therefore, these data suggest that CT functions to increase the number of OT-II specific T cells when co-administered with OVA, without altering the migration pattern of OVA-specific T cells *in vivo*.

### CT does not Alter the Rate of Antigen-specific Cells Dividing in Response to Orally Administered Antigen Stimulation in vivo

The increase in GFP^+/−^ OT-II T cell number observed in the PP and the MLN after co-administration of OVA with CT compared with OVA alone, could be due to either increased number of T cells undergoing cellular division, an equivalent number of cells undergoing more rounds of cellular divisions in response to the antigen or be a consequence of enhanced migration of GFP^+/−^ OT-II T cells into the MLN. Since CT did not alter cellular migration patterns *in vivo* (Figure 1), the effect of orally administered CT on the *in vivo* kinetics of antigen-specific T cell division was determined.

**Figure 2:**
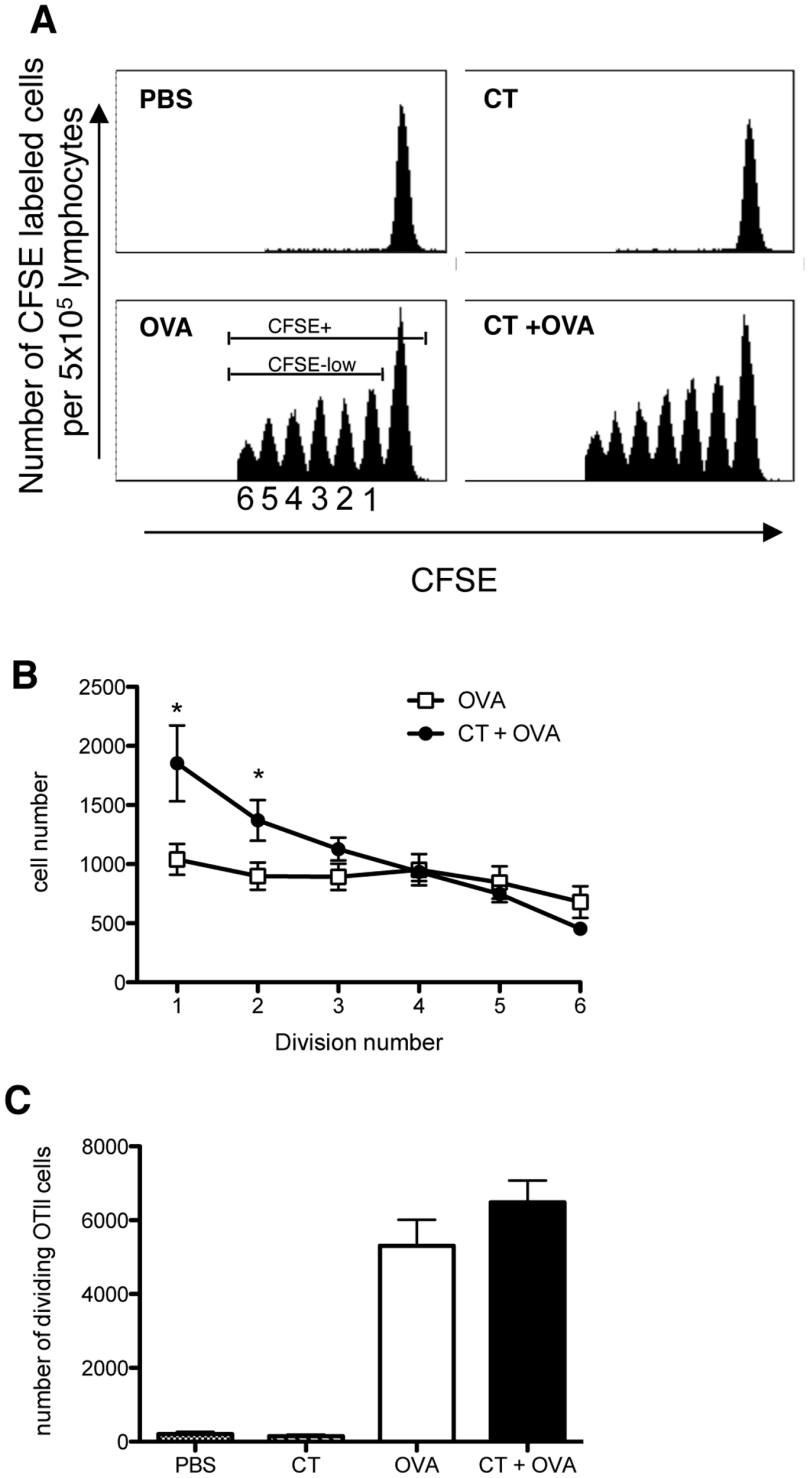
CT induces an increase in the number of dividing OT-II cells without altering the number of cellular divisions in the draining MLN. CFSE-labeled OT-II cells (5×10^6^) were i.v. injected into recipient C57BL/6 mice. One day later, mice were orally immunized with either PBS (n = 3), 10 µg CT (n = 3), 15 mg OVA (n = 10) or 10 µg CT with 15 mg OVA (n = 10). Five days after immunization, MLN were removed and dilution of CFSE from OT-II cells as a measurement of cellular division was analyzed using flow cytometry. Propidium iodide was used to exclude dead cells from the analysis. A: Shown is a representative histogram from one individual animal of each group. B: Mice were analyzed for the total number of CFSE-labeled CD4^+^ T cells within each cellular division. Shown are OVA fed (filled squares) and CT with OVA fed (filled circle) mice. Data is represented as the mean ± SEM of each group of mice (n = 10). *denotes *P*<0.05, Students’ t-test. C) The total number of CFSE-labeled, viable CD4^+^ T cells per 5×10^5^ lymphocytes is shown. All data are representative of 2 independent experiments and represented as the mean ± SEM of each group of mice.

CFSE-labeled OT-II cells were adoptively transferred to recipient C57BL/6 mice and one day later mice were orally immunized with either PBS, 10 µg CT, 15 mg OVA or 10 µg CT and 15 mg OVA. The location of OT-II cells and dilution of CFSE dye as a marker of the number of cellular divisions was analyzed by flow cytometry five days after immunization (Figure 2). A representative histogram of CFSE-labeled OT-II cells cells in the MLN of immunized mice from each group is depicted in Figure 2A. A single peak of undivided CFSE-labeled OT-II cells was observed in MLN (Figure 2A) and PP (data not shown) after immunization of mice with CT or PBS. No additive effect of co-administration of CT to OVA antigen was noted in the MLN, compared to OVA alone, as CFSE-labeled OT-II cells had undergone 6 rounds of cellular division in each treatment group (Figure 2A). This data suggested that CT did not alter the rate of cellular division of antigen-specific T cells.

To further analyze the effect of CT on antigen-induced proliferation of T cells, the number of CFSE-labeled cells per dividing peak located within the MLN was quantified (Figure 2B). A greater number of CFSE-labeled OT-II cells were contained within the first and second peak of division of mice fed CT with OVA compared to mice that were fed OVA alone (P<0.05) (Figure 2B), suggesting that CT administered with antigen increased the number of dividing antigen-specific T cells. Furthermore, when analyzing the total number of dividing (CFSE low) OT-II cells present in the MLN of OVA and CT with OVA-immunized mice, we noticed an increase in the number of dividing CFSE-labeled OT-II cells in the latter group (Figure 2C), suggesting that CT co-administered with OVA increased the number of dividing antigen-specific T cells *in vivo.* Indeed, these findings further support the data shown in figure 1, and suggest that CT increased the number of antigen-specific T cells without altering the kinetics of OT-II cell division.

Moreover, it has been reported that oral immunization with CT+OVA induces the expression of α4β7 integrin on OT-I CD8^+^ T cells [36]. Therefore, we wished to examine the expression of α4β7 integrin on CFSE-labelled CD4^+^ OT-II cells in the MLN, PP and spleen 5 days post immunization. We identified that CFSE-labelled CD4^+^ OT-II from PBS, CT, OVA and CT+OVA immunized animals were negative in all organs for the expression of α4β7 at 5 days post immunization. Hence, we concluded that administration of CT with OVA does not alter the migration pattern of CD4^+^ T cells, and does not induce the expression of α4β7 at 5 days after immunization.

### Intranasal Administration of CT and OVA Increased the Total Number of Antigen-specific T Cells in vivo

Since CT is also used as an adjuvant administered via the i.n. route [37], [38], this study continued to examine the adjuvant effect of i.n. administered CT on antigen-specific T cells. First, the optimal volume that can be administered i.n. with minimal amount of fluid entering the gastrointestinal and/or lower respiratory tract was determined, using Evans Blue dye in increasing volumes. Careful monitoring of the location of dye *in situ* revealed that a 12 µl volume dose remained in the upper respiratory tract and was absent from the gastric tissue, and this volume was used for all subsequent i.n immunization studies (Table 1). Subsequently, the minimum amount of OVA administered i.n. to B6.OT-II-recipient mice was titrated and 25 µg was selected as the optimal dose, based on the observation that proliferation of CFSE-labeled OT-II cells was detected in all animals immunized with this dose of OVA (data not shown).

**Table 1:** Distribution of Evans Blue dye after i.n. administration.

	12 µl Evans Blue				20 µl Evans Blue			
	Time after inoculation				Time after inoculation			
LOCATION	0 h	1 h	4 h	22 h	0 h	1 h	4 h	22 h
nasal cavity	+++^a^	+++	+++	+++	+++	+++	+++	+++
lung	− − −	+	− − −	− − −	+++	+++	+++	+++
lung and stomach	− − −	++	+++	− − −	− − −	+++	+++	− − −

a + indicates the presence of dye, in relative amounts, - indicates its absence from the organ.

**Figure 3:**
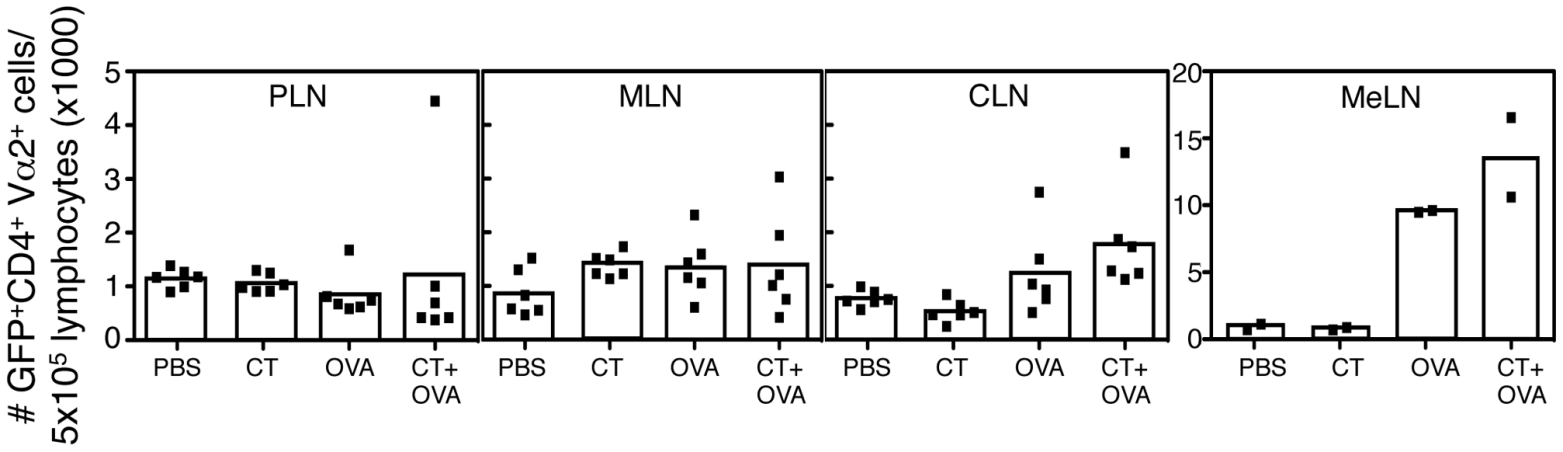
Intranasal administration of CT with OVA increases the number of GFP OT-II cells in draining lymph node. GFP^+/−^ OT-II cells were injected into C57BL/6 mice. One day later, mice were i.n. immunized with PBS, 5 µg CT, 5 µg CT with 25 µg OVA or 25 µg OVA alone. Five days after immunization, the number of GFP^+/−^ OT-II (GFP, Vα2, CD4^+^) cells in the PLN, MLN, cervical lymph nodes (CLN) and mediastinal lymph nodes (MeLN) were enumerated by flow cytometry. Shown are the number of cells detected in the PLN, MLN and CLN of individual mice (symbols). The MeLN of groups of 3 mice were pooled (each symbol representing a pool of 3 mice). The columns indicate the average of each treatment group. Data were obtained from 2 individual experiments. *denotes P<0.05 (one way ANOVA).

To examine the effect of i.n. administered CT on antigen-specific T cells numbers *in vivo*, GFP^+/−^ OT-II lymphocytes were adoptively transferred to recipient C57BL/6 mice. One day later, mice were i.n. immunized with either PBS, 5 µg CT, 5 µg CT with 25 µg OVA or 25 µg OVA in a 12 µl volume. Mice were euthanized 1 and 5 days after i.n. immunization and the number of GFP^+/−^ OT-II T cells within each organ was enumerated by flow cytometry (Figure 3). One day following immunization, an equivalent number of GFP^+/−^ OT-II cells were located within the peripheral lymph node (PLN, i.e. brachial), cervical lymph node (CLN), MLN and mediastinal lymph nodes (MeLN, pooled groups of 3 mice) of all mice (data not shown).

At five days after i.n. immunization, no differences were observed in the number of GFP^+/−^ OT-II cells in the PLN and MLN between various treatment groups (Figure 3). In the CLN, significantly more (P<0.05, one way ANOVA) GFP^+/−^ OT-II cells were detected in mice treated with CT and OVA compared with the other treatment groups (Figure 3). A similar trend was observed in the MeLN, where increased numbers of GFP^+/−^ OT-II were detected following treatment with CT and OVA or OVA alone compared with PBS or CT treated mice (Figure 3). The analysis of nasal associated lymphoid tissue (NALT) from all groups of animals revealed negligible numbers (<100) of OT-II T cells present within this compartment, therefore no further analysis of OT-II cells in the NALT was performed in this study. Thus, the findings of this experiment suggested that similar to oral administration, CT administered via the i.n. route increased the total number of antigen-specific T cells in the draining MeLN and the CLN but not at other sites.

### The Number of CFSE-labeled OT-II Cells Increased Following i.n. Administration of CT and OVA without Altering the Kinetics of Cellular Divisions

**Figure 4:**
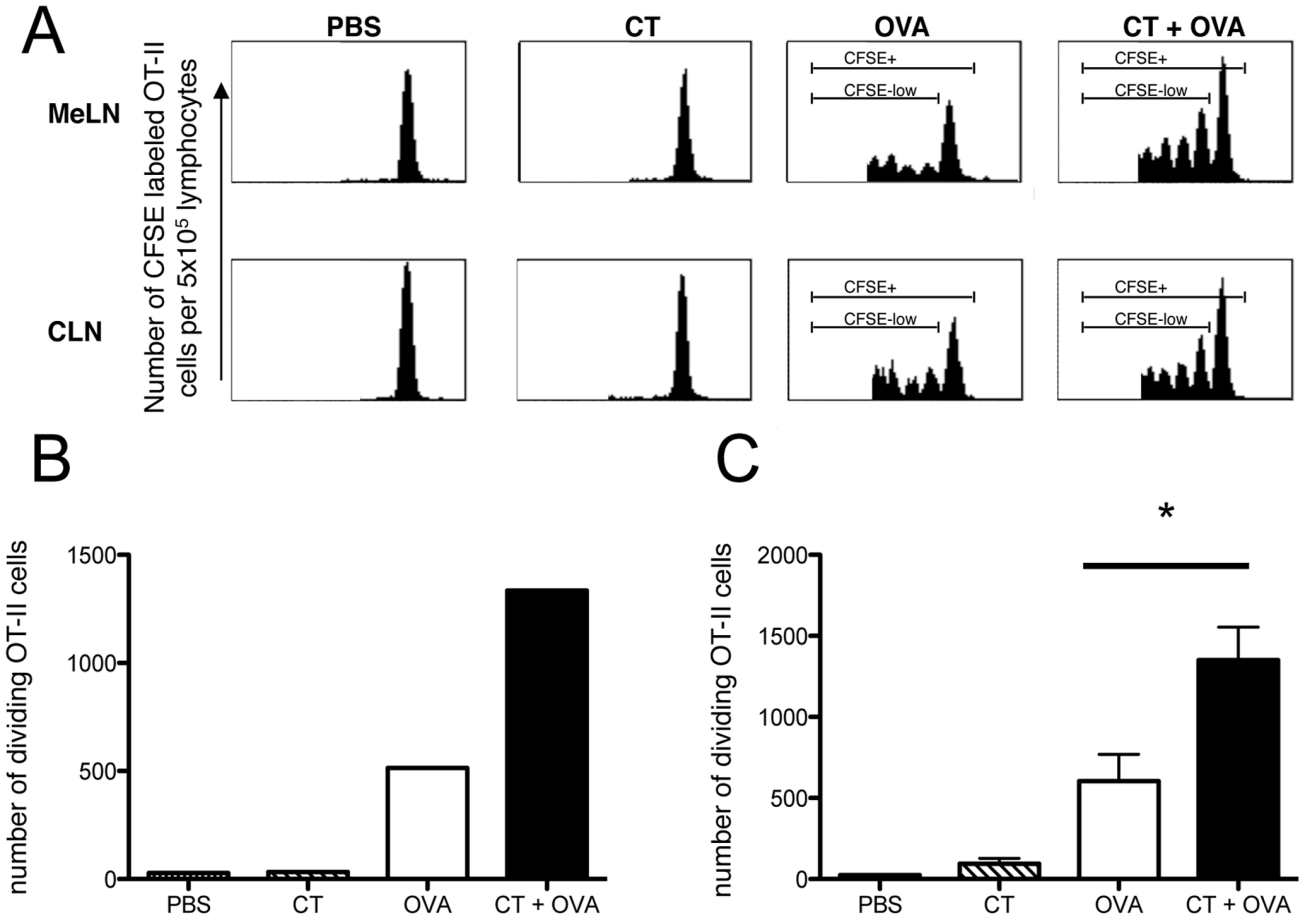
Intranasal administration of OVA with the adjuvant CT results in an increased proliferation of CFSE labeled OT-II cells *in vivo*. CFSE -labeled OT-II cells (5×10^6^) were adoptively transferred into C57BL/6 mice. One day after transfer, mice were i.n immunized with either PBS, CT, OVA or CT with OVA (n = 3 per group). Five days after immunization, MeLN (pooled), CLN and MLN were removed and analyzed using flow cytometry to determine the presence and number of divisions undergone by CFSE OT-II cells *in vivo*. Propidium iodide (PI) was used to exclude dead cells from the analysis. (A) Shown is a representative histogram from one individual animal of each group. The number of dividing CFSE-labeled, PI negative, Vα2^+^, CD4^+^ T cells in the MeLN (B) and CLN (C) was determined per 5×10e^5^ lymphocytes. Data are representative of 3 individual experiments. *denotes *P*<0.05, Students’ t-test.

To determine whether the increased number of OT-II cells present in the draining MeLN of CT and OVA i.n immunized mice was due to enhanced local proliferation, or an alteration of the kinetics of division, we analyzed the migration and proliferation pattern of CFSE-labeled OT-II cells using flow cytometry five days following i.n. immunization (Figure 4). Transgenic OT-II cells were labeled with the fluorescent dye CFSE and adoptively transferred into C57BL/6 mice. The following day, mice were i.n. immunized with PBS, 5 µg CT, 5 µg CT with 25 µg OVA or 25 µg OVA alone. One and five days after immunization, cellular suspensions of secondary lymphoid organs were analyzed by flow cytometry to determine the location and the level of proliferation of antigen specific T cells. One day after i.n. immunization, no proliferation of OT-II lymphocytes was observed in any of the tissue analyzed (data not shown). Five days after immunization, dividing CFSE-labeled cells were detected in the CLN and MeLN of the OVA and CT with OVA immunized mice, and at both sites the OT-II cells had undergone 4 detectable rounds of cellular division (Figure 4A). No proliferation was detected in the CLN and MeLN of CT and PBS immunized mice. In addition, no dividing CFSE-labeled cells were detectable in the MLN and PLN (data not shown) of any mice, irrespective of the vaccination, suggesting that little or no antigen had entered the gastrointestinal tract and dividing cells had not re-circulated.

We next performed further analysis of the total number of CFSE-labeled OT-II cells that had divided in the MeLN and CLN of mice i.n immunized with OVA or CT with OVA 5 days post vaccination (Figure 4B, 4C). In comparison to OVA-immunized mice, there was a greater than 2 fold increase in the number of proliferating OT-II cells in the MeLN and CLN (*P*<0.05) of CT with OVA immunized mice, suggesting that i.n administered CT increased the number of antigen-specific T cells in draining LN through increased number of proliferating cells.

### CT does not Alter the Kinetics of Cellular Division of CFSE-labeled OT-II Cells Stimulated with Peptide Pulsed DCs in vitro

The results from this study suggested that orally or i.n. administered CT increased the number of antigen-specific T cells and did not alter the number of cellular divisions of antigen-specific T cells detected at a particular timepoint *in vivo*. There are several parameters that cannot be controlled when performing *in vivo* studies, the key one being migration of stimulated antigen-specific T cells from one site to another. To eliminate the possibility that activated, CFSE-labeled cell migration out of the MLN or MeLN in immunized mice affected the findings of the experiment, an *in vitro* experiment was performed to determine if CT alters the kinetics of cellular division.

**Figure 5:**
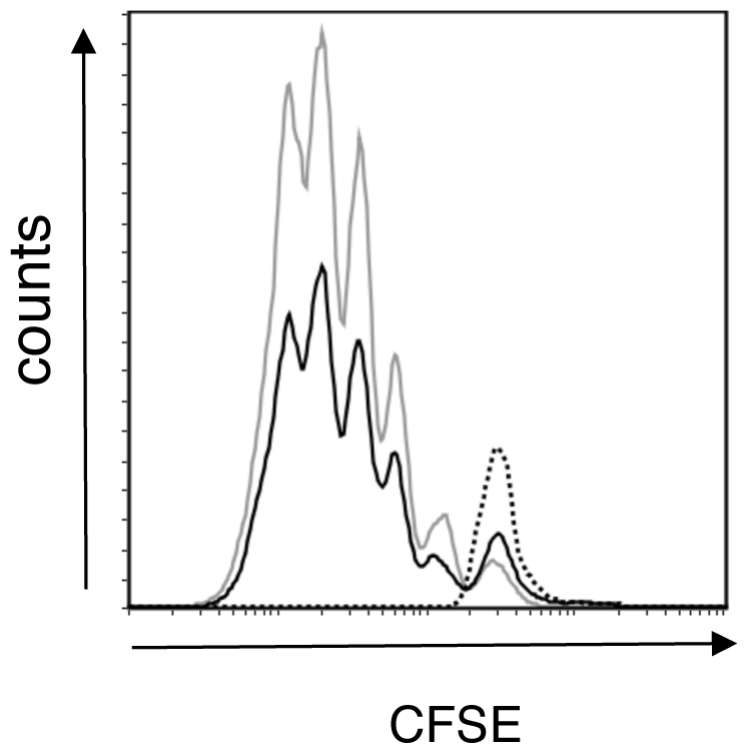
Increased proliferation of OT-II cells stimulated with CT and OVA pulsed BMDCs. BMDC were pulsed with CT or media (dashed line), OVA peptide (black line) or OVA peptide with CT (grey line) and cultured with CFSE-labeled OT-II lymphocytes for 4 days. Lymphocyte proliferation was determined using flow cytometry. PI was used to exclude dead cells from the analysis. The flow cytometry plots represent cells harvested from triplicate cultures. Data are representative of 2 independent experiments.

Bone marrow derived dendritic cells (BMDCs) were pulsed with either 1 µg OVA_323–339_ peptide or with the same amount of OVA_323–339_ peptide mixed with CT, and were cultured for 4 days with CFSE-labeled OT-II cells. Flow cytometry was used to analyze BMDC-induced cellular division of the CFSE-labeled OT-II (Figure 5). BMDC pulsed with CT alone or media did not induce proliferation of CFSE-labeled OT-II cells, indicating any detected cellular division was antigen driven. Stimulation with OVA_323–339_ peptide induced 5 rounds of division (Figure 5). Incubation of BMDC with CT and OVA_323–339_ increased the total number of dividing CFSE-labeled OT-II cells, indicated by the larger peaks at each stage of cellular division (Figure 5). Comparable results were obtained at days 2 and 3 post stimulation, indicating that cells were not dividing faster at any timepoint analyzed (data not shown). Similarly, an increased number of proliferating OT-II cells but no difference in number of cellular divisions was observed in cultures in which BMDC were pulsed with OVA or with CT and OVA (data not shown).

## Discussion

In this study, adoptive transfer of naïve, fluorescently-labeled transgenic lymphocytes was used to model the behavior of antigen-specific T cells *in vivo* following immunization with CT and antigen. To trace the adoptively transferred lymphocytes, cells were either labeled with the intracellular fluorescent dye CFSE, or were obtained from GFP transgenic animals. The fluorescent dye CFSE is a widely used immunological tool enabling the monitoring of cellular division and migration pattern of lymphocytes *in vivo* [39], [40]. However, during periods of extensive stimulation and cellular division of CFSE-labeled lymphocytes, fluorescence may be lost due to exhaustion of the fluorochrome. Unlike CFSE-based fluorescence, GFP-based fluorescence is not lost following division of cells. In this study, we used heterozygous B6.GFP^+/−^ mice [26], as homozygous GFP mice exhibit growth retardation and die at 4 to 6 weeks of age due to unknown causes [41] were mated to homozygous B6.OT-II mice to generate B6.OTII^gfp+/−^ mice.

The first aim of this study was to determine the effect of CT on antigen-specific T cell migration. Initially, GFP OT-II lymphocytes were adoptively transferred into C57BL/6 recipients to examine the effect of CT on the migration of antigen-specific T cells. Administration of CT alone to GFP OT-II recipient animals did not alter the migration pattern of antigen specific CD4^+^ T cells, which were only detectable in secondary lymphoid tissues. This data suggests that the mucosal adjuvant CT, in the absence of antigen, does not alter the migration pattern of naïve antigen specific CD4^+^ T cells compared to PBS controls.

Naïve lymphocytes are programmed to continuously recirculate from blood through secondary lymphoid tissue to blood [42] for the duration of their lifespan, assuming they do not come in contact with APCs presenting antigen [43]. Therefore naïve lymphocytes migrate only to secondary lymphoid organs but memory T cells migrate to non-lymphoid organs [44]. Our findings correlate with previous work by Sydora *et al*. who reported the inability of naïve lymphocytes obtained from secondary lymphoid tissues to enter the IEL compartment of the gut after adoptive transfer [45].

Further studies focused on the effect of co-administration of CT with antigen, via the oral or i.n route, on the migration and division pattern of adoptively transferred GFP^+/−^ OT-II cells. Based on previous findings by Kato *et al.*, it was important to minimize the amount of OVA administered to mice, since high oral doses of OVA are known to induce unresponsive OVA-specific T cells in mucosal inductive sites [46]. Furthermore, it has been established that i.n. immunization is more efficient, as it requires lower doses of antigen to induce T cell proliferation compared with oral immunization [3], [37], [46]. This study then examined the effect of mucosally administered CT and antigen on CD4^+^ T cell numbers. The results from this study identified that in comparison with OVA alone, co-administration of CT and OVA via the oral route significantly increased the number of GFP^+/−^ OT-II cells in the MLN and PP 5 days after immunization. Similarly, 5 days after i.n. immunization, the number of GFP^+/−^ OT-II cells was increased in the MeLN of mice immunized with CT and OVA. In addition to draining LN, the NALT is also recognized as an important site for induction of mucosal immune responses following intranasal administration of antigen. However, our analysis of NALT following i.n. immunization revealed negligible numbers of OT-II T cells present within this compartment, irrespective of the immunization treatment, and therefore no conclusions can be drawn on the effect of CT administration on antigen specific T cell activation in the NALT using this technique.

Our observations suggest that CT functions as a mucosal adjuvant by increasing the number of antigen-specific CD4^+^ T cells in immunized mice. This increase in antigen-specific T cell numbers after immunization of antigen with CT has been reported by others [14], [22]. Hornquist *et al.* reported a 20 to 40 fold increase in the frequency of primed antigen-specific T cells after oral immunization with CT and keyhole limpet hemocyanin (KLH) compared with mice immunized with KLH alone [14]. Taken together, findings from this study and others highlight that co-administration of antigen with CT increases the number of antigen-specific T cells *in vivo.*

It was hypothesized that the CT-induced increase in antigen-specific CD4^+^ T cell numbers could be due to 1) equivalent numbers of cells undergoing more rounds of cellular divisions in response to the antigen, 2) the induction of more T cells to divide, or 3) increased migration of antigen-specific T cells to a site of antigen exposure. To address these hypotheses, adoptive transfer of CFSE-labeled OT-II cells was employed, enabling the visualization of cellular division *in vivo*. A significant increase in CD4^+^ T cell numbers was evident due to the co-administration of CT and OVA in the draining lymph node of i.n or orally immunized animals. In addition, our data showed that CT did not alter the number of cellular divisions undergone by antigen-specific CD4^+^ T cells *in vivo* at this timepoint, as the CFSE division profile was similar in both treatment groups. As the findings from these studies were not definitive, an *in vitro* study was performed to elucidate the effect of CT on the kinetics of cellular division without cellular migration affecting the outcome of the experiment. Indeed, these *in vitro* experiments confirmed our *in vivo* observations and no effect of CT on the number of cellular divisions or the kinetics of division was noted. Whether CT has a similar effect when co-administered with a different antigen remains to be determined. Furthermore, as our studies did not examine apoptosis rates of antigen-specific CD4^+^ T cells, the contribution of CT-induced apoptosis of antigen specific CD4^+^ T cell number *in vivo* remains to be eluded.

It has been previously shown that CT+OVA induces the expression of α4β7 integrin on OT-I CD8^+^ T cells, which ultimately effects the migration pattern of CD8^+^ T cells *in vivo* [36]. In our study, we did not detect any expression of α4β7 integrin on OT-II CD4^+^ T cells 5 days post oral immunization with CT+OVA. It should be noted that the studies examining α4β7 integrin expression on CD8^+^ T cells used twice as much CT and OVA compared to the amounts used in our study, and this may have an effect on the levels of α4β7 expression observed. Further studies need to be performed to determine the effect of oral immunization with CT+OVA on α4β7 integrin expression by CD4^+^ T cells *in vivo*.

Overall, this study identified that the adjuvant effect of CT on antigen-specific CD4^+^ T cells is not due to altering the pattern of cellular migration of antigen-specific CD4^+^ T cells into other non-mucosal effector sites, nor due to a modification of the kinetics of cellular division as demonstrated in a controlled *in vitro* system, but immunization with CT affects immune responses by increasing the number of CD4^+^ antigen specific T cells. So how does CT increase the number of antigen specific T cells when co-administered with antigen? CT is known to facilitate luminal antigen transport across the epithelium by increasing gut permeability, leading to increased access of antigen to the immune system [10]. DCs in the underlying mucosa can move in response to enterotoxin adjuvants that enter via M cells [47]. In addition, DCs can open tight junctions between epithelial cells, and use dendrites to directly sample antigen from outside the epithelium [48]. The DC can undergo phenotypic and functional maturation [49], upregulating membrane molecules important for T cell stimulation such as MHC antigens, B7.1, B7.2, adhesion molecules and receptors for chemokines [50]. This process allows them to exit inflamed tissue and to migrate to draining lymph nodes to prime naïve T cells. Anjuere *et al.* identified that DCs isolated from the MLN of mice orally immunized with CT were potent activators of naïve T cells, could be transferred to naïve animals and induced a Th2 immune response [51]. Similarly, CT can activate DCs when administered via the i.n. route. Porgador *et al*. identified NALT DCs are the predominant APC involved in the induction of immunity after i.n. co-administration of OVA and CT, and that DCs isolated from the CLN or NALT of i.n. immunized mice could stimulate OVA-specific T cells *in vitro* [52].

Furthermore, conventional DCs are essential for the priming of antigen-specific CD4^+^ T cell responses during vaccination with antigen and CT [20]. This may be due to the direct effect that CT has on DCs, eg. inducing their maturation, and/or increasing the expression of HLA molecules, costimulatory molecules and functional CCR7 and CXCR4 chemokine receptors, which may render DCs responsive to migratory stimuli towards secondary lymphoid organs [23]–[25]. CT also promotes DCs to drive Th2 responses by inhibiting the production of IFN-g, IL-12 and TNFα [23], [53]–[55], by enhancing ICAM-1 expression and the production of cytokines such as IL-10 and IL-6, which are important for Th2 cell differentiation [56], as well as through the induction of a population of IL-10 producing regulatory T cells [56].

The findings reported here suggest that CT increases the number of OVA-specific CD4^+^ T cells *in vivo* via an indirect effect, possibly via antigen presenting cells, such as the dendritic cell. Dendritic cells reside in the NALT, Peyer’s patches and MLN where they can easily access foreign antigen and can be affected by adjuvants such as CT [51], [52]. In this study, the direct effect of CT on activation and migration of dendritic cells *in vivo* was not analyzed, and future work examining this area may identify the mechanism whereby CT functions as a mucosal adjuvant. In addition, to further understand effects of CT on CD4^+^ T cells responses and improve vaccine design, studies investigating in detail the longevity memory T cell responses are also warranted.
